# Supplementary material for: Splice-Junction-Based Mapping of Alternative Isoforms in the Human Proteome
Source: Cell Rep. Author manuscript; Available in PMC 2020 Jan 15. (PMC6961840; doi:10.1016/j.celrep.2019.11.026)
Supplement: 2 [file NIHMS1546469-supplement-2.zip › DF1/PXD006675/RightAtrium/RightAtrium_53_RBBP4_GPDAVEERVINEEYK.pdf]

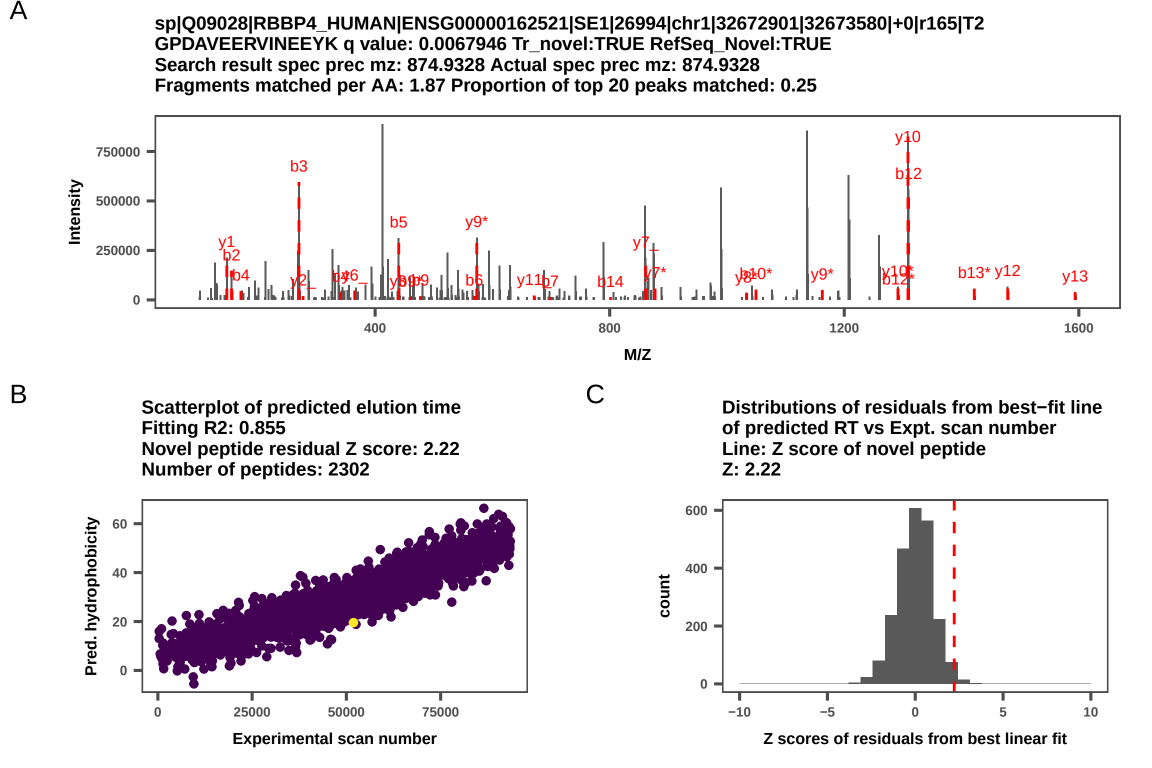
A
sp|Q09028|RBBP4_HUMAN|ENSG00000162521|SE1|26994|chr1|32672901|32673580|+0|r165|T2
GPDAVEERVINEEYK q value: 0.0067946 Tr_novel:TRUE RefSeq_Novel:TRUE
Search result spec prec mz: 874.9328 Actual spec prec mz: 874.9328
Fragments matched per AA: 1.87 Proportion of top 20 peaks matched: 0.25
Intensity
750000
500000
250000
0
M/Z
400
800
1200
1600
y1
b2
b4
b3
y2
y6
b5
y9*
b6
y11
b7
b14
y7
y7*
b10*
y9*
y10
b12
y10*
b12*
b13*
y12
y13
B
Scatterplot of predicted elution time
Fitting R2: 0.855
Novel peptide residual Z score: 2.22
Number of peptides: 2302
Pred. hydrophobicity
60
40
20
0
0
25000
50000
75000
Experimental scan number
C
Distributions of residuals from best−fit line
of predicted RT vs Expt. scan number
Line: Z score of novel peptide
Z: 2.22
count
600
400
200
0
−10
−5
0
5
10
Z scores of residuals from best linear fit
